# Inhibition of NR2F2 suppresses invasion ability and modulates EMT marker in head and neck squamous cell carcinoma

**DOI:** 10.1007/s12672-025-03539-3

**Published:** 2025-10-15

**Authors:** Joo Kyung Noh, Seon Rang Woo, Min Kyeong Lee, Jung Woo Lee, Young Chan Lee, Seong-Gyu Ko, Young-Gyu Eun

**Affiliations:** 1 Department of Biomedical Science and Technology, Graduate School, Kyung Hee University, Seoul, Republic of Korea; 2 Department of Otolaryngology-Head & Neck Surgery, Kyung Hee University School of Medicine, Kyung Hee University Medical Center, #1 Hoegi-dong, Dongdaemun-gu, Seoul, 130-702 Republic of Korea; 3 Department of Oral and Maxillofacial Surgery, School of Dentistry, Kyung Hee University, Seoul, Republic of Korea; 4 Department of Preventive Medicine, College of Korean Medicine, Kyung Hee University, Seoul, Republic of Korea

**Keywords:** NR2F2, Head and neck squamous cell carcinoma, Migration, Invasion, Epithelial mesenchymal transition

## Abstract

The nuclear receptor subfamily 2 group F member 2 (NR2F2) has emerged as a potential therapeutic target in cancer due to its involvement in tumor progression, metastasis, angiogenesis, and epithelial-mesenchymal transition (EMT). However, its precise role in head and neck squamous cell carcinoma (HNSCC) and other cancer types remains to be elucidated. To investigate the role of NR2F2 in HNSCC, we examined its expression in relation to lymph node metastasis (LNM) in patient samples. Further functional studies were conducted using knockdown experiments and NR2F2 inhibitors, COUP-TFII inhibitor A(CIA) and 4-methoxynaphthalene-1-ol(4-MNol), in HNSCC cell lines. Invasion and migration assays were performed to assess the impact of NR2F2 modulation. Additionally, Western blot analysis was conducted to investigate the effect of NR2F2 inhibition on epithelial-mesenchymal transition (EMT) markers. NR2F2 expression was found to be upregulated in HNSCC patients with LNM, suggesting its involvement in tumor progression and metastasis. Knockdown of NR2F2 significantly decreased the invasion and migration of HNSCC cells. Inhibition of NR2F2 using CIA and 4-MNol also resulted in reduced invasion ability and modulation of EMT markers in HNSCC cells.

This study highlights the significance of NR2F2 in HNSCC and provides evidence for its role in promoting the invasion and migration of cancer cells. The findings suggest that NR2F2 may serve as a potential therapeutic target in HNSCC and other cancer types. Further research and development of NR2F2-targeted therapies are warranted for improved treatment strategies in cancer patients.

**Supplementary Information:**

The online version contains supplementary material available at 10.1007/s12672-025-03539-3.

## Introduction

Head and neck squamous cell carcinomas (HNSCCs) are the most prevalent malignancies in the head and neck region, originating from the mucosal epithelium of the oral cavity, pharynx, and larynx [1]. HNSCC is a globally widespread malignancy, with over 790,000 new cases and 450,000 deaths reported each year worldwide [2]. The standard management of HNSCC typically involves a combination of surgery, radiotherapy, and neoadjuvant or adjuvant chemotherapy [3]. However, patients with locally advanced HNSCC often face a challenging prognosis, as disease recurrence occurs in approximately 40–50% of cases despite modern multimodality treatments [4]. This highlights the urgent need for treatment options that can improve overall survival or allow for a less aggressive standard of care [5].

Metastasis and therapy resistance present significant clinical challenges in cancer management [6]. The acquisition of migratory and invasive characteristics by cancer cells is crucial for their ability to spread to distant sites and invade adjacent tissues [7]. Lymph node invasion is a common feature of HNSCC and serves as a strong predictor of patient mortality [8]. The process of HNSCC oncogenesis involves a complex interplay of various pathways and processes contributing to tumor cell invasion and metastasis.

Nuclear receptor subfamily 2 group F member 2 (NR2F2), a member of the Chicken ovalbumin upstream promoter transcription factor (COUP-TF) family, plays a critical role in cancer progression [9]. It is highly conserved and expressed in the mesenchymal tissues of various organs that depend on mesenchymal-epithelial interactions [10, 11]. NR2F2 has been shown to significantly impact tumor growth and metastasis by regulating tumor angiogenesis [12]. Furthermore, studies have explored the regulatory function of NR2F2 in stemness. It is highly expressed in differentiated and adult mesenchymal cells, affecting terminal cell differentiation [13, 14].

In skin squamous cell carcinoma (SCC), NR2F2 is uniquely expressed in malignant SCC compared to benign tumors [15]. Studies have demonstrated that NR2F2 is essential for promoting the malignant tumor state, controlling epithelial-mesenchymal transition (EMT), and invasive features. Insulin stimulation has been shown to increase NR2F2 expression, promoting cell invasion and migration accompanied by changes in EMT-related molecular markers in breast cancer [16]. NR2F2 expression was positively correlated with cell invasion and migration. However, the role of NR2F2 in regulating the migration and invasion properties of HNSCC cells has not been previously reported. Therefore, we aim to investigate whether NR2F2 can modulate the migration and invasion of HNSCC cells by altering the expression of EMT markers.

In this study, we will examine the effects of NR2F2 on HNSCC cell migration and invasion. We will manipulate NR2F2 expression using genetic techniques, including loss of function experiments in vitro. By elucidating the role of NR2F2 in HNSCC cell migration and invasion, we sought to gain a better understanding of the molecular mechanisms underlying the aggressive behavior of HNSCC and identify potential therapeutic targets for intervention.

## Materials and methods

### Patients and cohorts

The Cancer Genome Atlas (TCGA) cohort data were downloaded from the UCSC Cancer Genomics Browser (htttp://xena.ucsc.edu/). Clinical information of 520 HNSCC was downloaded from the TCGA database (https://gdc.cancer.gov/, assessed October 2020). Vanderbilt (GSE3292) [17] and UNC (GSE39368) [18] datasets were downloaded from the National Center for Biotechnology Information Gene Expression Omnibus database (https://www.ncbi.nlm.nih.gov/geo/). Table 1 shows the clinical and pathological characteristics of each cohort.

**Table 1:** HNSCC patient’s information of TCGA, vanderbilt, and UNC cohort

	TCGA	Vanderbilt	UNC
**Number of patients**	520	36	138
**Gender**			
Male	383	29	95
Female	134	6	43
**Age**			
>=60 years old	286	12	61
< 60 years old	233	23	77
**Primary tumor**			
T1	51		13
T2	158		27
T3	121		25
T4	189		52
**Regional lymph node**			
N0	221		51
N1	79		15
N2	205		46
N3	11		5
**Stage**			
I	27	2	8
II	84	3	14
III	94	9	28
IV	315	22	84
**HPV status**			
Positive	68	8	14
Negative	452	24	82
**Smoking**			
Yes	384	32	105
No	136	4	33
**Alcohol**			
Yes	347	22	50
No	162	14	88

### Chemicals

COUP-TFII inhibitor A (CIA) was from ChemBridge (7960292, ChemBridge, CA, US) [19]. 4-methoxynaphthalene-1-ol (4-MNol) was purchased from Sigma-Aldrich (174556, Sigma-Aldrich, Missouri, US) [20].

### Cell culture

The human HNSCC cell line HSC3-M3 was purchased from the Japanese Collection of Research Bioresources Cell Bank. YD38 and YD8 were purchased from the Korean Cell Line Bank. HSC3-M3 cells were cultured in EMEM containing 10% heat-inactivated fetal bovine serum (FBS, Hyclone) and 1% penicillin-streptomycin (PS, Corning Inc.). YD38 and YD8 cells were cultured in Rosewell Park Memorial Institute-1640 medium containing 10% FBS and 1% PS. All cell lines were maintained at 37 °C in a humidified incubator containing 5% CO_2_.

### Transfection

Cells were transfected with negative control siRNA (Bioneer) or siNR2F2 (IDT) using Lipofectamine RNAiMAX Transfection Reagent (Thermo Fisher Scientific), according to the manufacturer’s instructions.

### Cell proliferation assay

Cell proliferation assay were performed in 96-well plates. Cells were plated at 5 × 103 cells/well in 100µL of culture medium. The next day, cells were transfected with siCON or siNR2F2. After 24 h, 48 h, and 72 h, cell viability was measured according to the manufacturer’s instructions (EZ-cytox, DoGenBio) and 10µL of the reagent was added to each well. After 2 h of incubation in a CO2 incubator, the conversion of the reagent into chromogenic formazan was evaluated with a spectrophotometer at 570 nm.

### Cell viability assay

HSC-M3, YD38, and YD8 cells were seeded in 96-well plates and treated with CIA (100, 1,000, 10,000, 100,000 nM). 5 × 10^3^ cells/well were seeded in 96-well plates. After 24 h of treatment with CIA, cell viability was measured according to the manufacturer’s instructions (EZ-cytox, DoGenBio) and 10µL of the reagent was added to each well. After 2 h of incubation in a CO2 incubator, the conversion of the reagent into chromogenic formazan was evaluated with a spectrophotometer at 570 nm.

### Western blotting

Cells were homogenized in RIPA buffer (1% Triton X-100, 1% sodium deoxycholate, 0.1% SDS, 150 mM NaCl, 50 mM Tris-HCl [pH 7.5], and 2 mM EDTA [pH 8.0]; Biosesang). Total protein was quantified using the BCA Protein Quantification Kit (Thermo Fisher Scientific) according to the manufacturer’s protocol. Equal amounts of protein and loading dye (5× SDS-PAGE loading buffer; iNtRON Biotechnology) were added per lane and resolved using an SDS-polyacrylamide gel. After electrophoresis, samples were transferred to polyvinylidene difluoride membranes (Millipore), which were then blocked for 1 h in 5% skim milk and TBS with 0.1% Tween-20 and incubated overnight at 4 °C with primary antibodies. The following antibodies were used: anti-NR2F2 (6434 S, Cell Signaling Technology), anti-actin (sc-47778, Santa Cruz Biotechnology), anti-ZO-1 (8193 S, Cell Signaling Technology), anti-claudin-1 (13255 S, Cell Signaling Technology), anti-N-cadherin (13116 S, Cell Signaling Technology), and anti-slug (9585 S, Cell Signaling Technology). Membranes were incubated with the corresponding secondary antibodies (Cell Signaling Technology) for 1 h at room temperature, followed by visualization using a chemiluminescence detection kit (RPN2232; GE Healthcare). All full-length, uncropped Western blot scans with molecular weight markers and lane labels are provided in Supplementary Figs. S6–S10.

### Transwell assay

Transwell assays to evaluate cell invasion were performed using a 24-well Transwell insert (3422, Corning Inc.) with an 8 μm pore polycarbonate membrane insert. Transwell inner chambers coated with 100µL of diluted Matrigel (volume of original Matrigel: FBS-free EMEM or RPMI1640 = 1:10) were used for the invasion assay, and these chambers were incubated at 37 °C for 4 h until the gel was set before use. 300µL of FBS-free EMEM or RPMI1640 with $\:3\times\:{10}^{4}$cells was added to the inner chamber, and 750µL of EMEM or RPMI1640 with 10% FBS was added to the outside wells. After incubation for 48 h for the invasion assay, cells that had invaded and were adhered to the lower surface were stained with hematoxylin and eosin. The cells were counted in four representative fields under light microscopy (200 x magnification).

## Results

### Expression of NR2F2 was upregulated in patients with lymph node metastasis

**Fig. 1:**
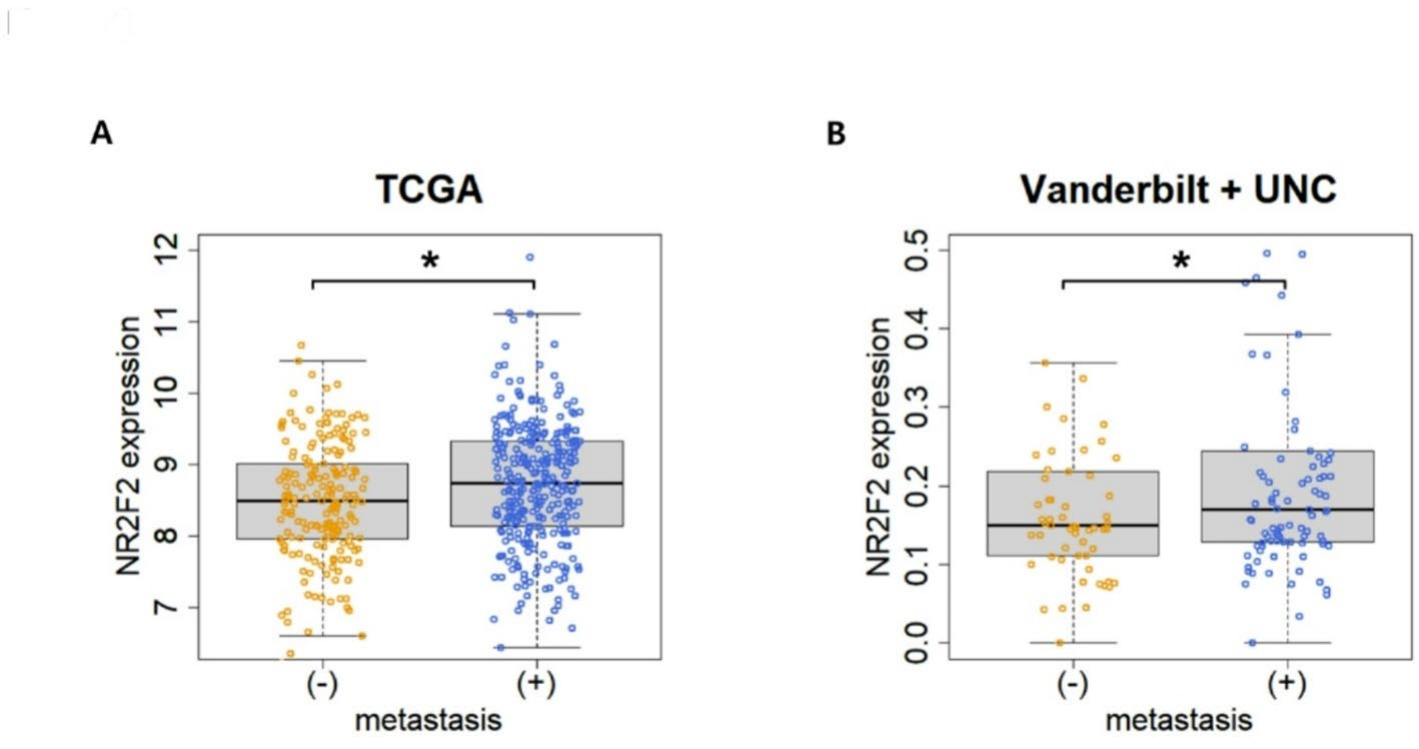
Comparison of NR2F2 expression between metastasis and non-metastasis groups in TCGA and Vanderbilt & UNC cohort. (A) The box plot shows the expression levels of NR2F2 in the TCGA cohort, with and without metastasis (*n* = 520, with metastasis; *n* = 323, without; *n* = 193, *p* = 0.004275). Statistical significance was calculated with a t-test. (B) The graph illustrates the difference in NR2F2 expression between metastasis and non-metastasis cases in the Vanderbilt + UNC cohort (*n* = 174, with metastasis; *n* = 94, without; *n* = 58, *p* = 0.02169). The asterisk mark indicates that the p-value is less than 0.05

We investigated the expression of NR2F2 in relation to lymph node metastasis (LNM) in HNSCC. In the TCGA cohort, which consisted of 323 samples with LNM and 193 samples without LNM. NR2F2 expression was significantly higher in the LNM group (Fig. 1A, *p* = 0.004275). Similarly, in the Vanderbilt & UNC cohort, which included 174 samples (with LNM; *n* = 94, without; *n* = 58), NR2F2 expression was also significantly elevated in the LNM group (Fig. 1B, *p* = 0.02169). These consistent findings highlight the strong association between elevated NR2F2 expression and the presence of metastasis.

### NR2F2 is associated with EMT and metastasis-related pathways in HNSCC

Analysis of NR2F2 expression and associated pathways in HNSCC revealed its connection to key processes underlying epithelial-mesenchymal transition (EMT) and metastatic potential (Supplementary Fig. 1A). Gene set enrichment analysis (GSEA) using TCGA HNSCC data ranked by NR2F2 correlation identified enrichment of gene sets related to regulation of supramolecular fiber organization (NOM p-value = 0.002008032), stress fiber assembly (Nominal p-value (NOM p-value) = 0.003960396), cell-matrix adhesion (NOM p-vauel = 0.0), and vascular endothelial cell proliferation (NOM p-value = 0.0). These pathways are functionally linked to cellular motility, cytoskeletal remodeling, and microenvironmental remodeling - hallmarks of EMT and metastasis. Additionally, enrichment of gene sets associated with ER stress (NOM p-value = 0.0) and protein catabolism (NOM p-value = 0.0) suggests activation of proteostatic stress responses, which can accompany cellular reprogramming events like EMT and the acquisition of stemness-like properties.

Consistent with the observed pathway-level associations, correlation analysis revealed a significant relationship between NR2F2 expression and canonical markers of epithelial-mesenchymal transition. Specifically, NR2F2 expression was negatively correlated with the epithelial marker CDH1 (*r* = -0.138, *p* = 0.00165), and positively correlated with the mesenchymal markers VIM (*r* = 0.397, *p* = 5.04 × 10^21^), ZEB1 (*r* = 0.606, *p* = 2.33 × 10^53^), and TWIST1 (*r* = 0.255, *p* = 3.93 × 10^9^) (Supplementary Fig. 1B).

These findings support a strong link between NR2F2 and a mesenchymal gene expression profile, consistent with its proposed role in promoting epithelial-mesenchymal transition in head and neck squamous cell carcinoma.

Taken together, the present data indicate that NR2F2 is associated with the regulation of key pathways and genes involved in cellular motility, cytoskeletal remodeling, and the EMT program in this cancer type.

### Knockdown of NR2F2 decreased the invasion ability of HNSCC cells

**Fig. 2:**
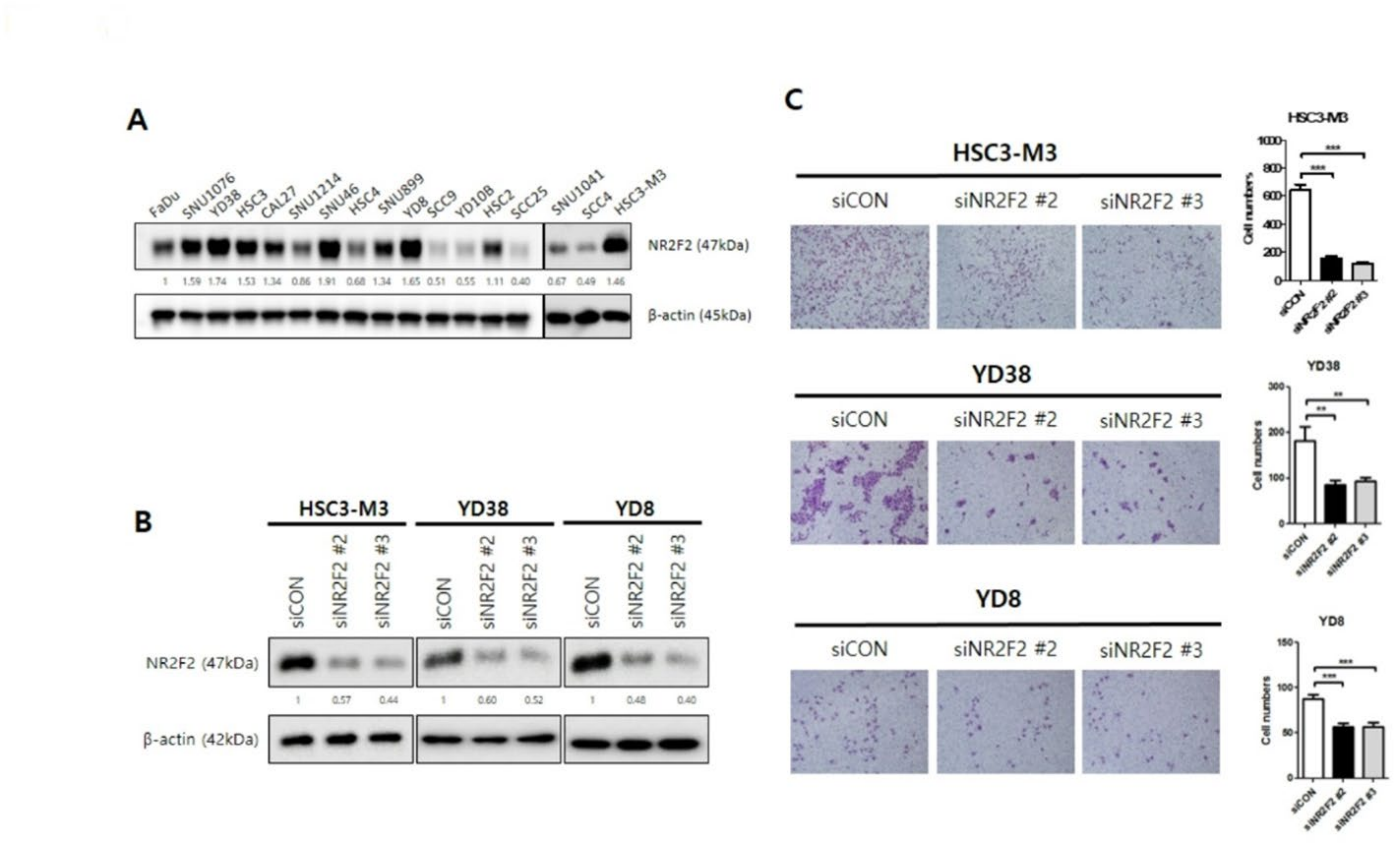
Knockdown of NR2F2 repressed invasion in HNSCC. (A) NR2F2 protein expression of 17 HNSCC cells was shown using western blot (WB). β-actin was loaded as a control. (B) Knockdown of NR2F2 using siRNA was determined to decrease the band in WB results in HSC3-M3, YD38, and YD8 cell lines. (C) Representative images of the cell invasion ability assay are shown. The effects of different treatments on the invasion ability of HNSCC cells were determined by counting the number of invading cells. Data are means $\:\pm\:$ SEM. (*n* = 4). Statistical significance was determined using a t-test. **p < 0.01; ***p < 0.005 vs. control. SEM, standard error of the mean

In order to investigate the effect of NR2F2 knockdown on invasion ability in HNSCC, we selected three cell lines (HSC3-M3, YD38, and YD8) that exhibited high NR2F2 expression out of 17 HNSCC cell lines tested (Fig. 2A). Using siRNA targeting NR2F2, we successfully knocked down NR2F2 expression and confirmed knockdown efficiency through western blot analysis (Fig. 2B and Supplementary Fig. 2). The representative image and quantification graph represent the assessed invasion ability of cells using the Transwell assay (Fig. 2C). Compared to the siCON-treated group, the siNR2F2-treated groups showed a significant decrease in the number of invaded cells. These findings provide evidence that the knockdown of NR2F2 can effectively reduce invasion ability in HNSCC.

To verify if the decrease in the number of invaded cells upon NR2F2 knockdown is not due to a decrease in proliferation, we performed a proliferation assay (Supplementary Fig. 3). Our results demonstrate that NR2F2 knockdown does not affect proliferation in YD38 and YD8 cells. Overall, our findings highlight the role of NR2F2 knockdown in reducing invasion ability in HNSCC cells and confirm that this effect is independent of proliferation.

### Inhibition of NR2F2 modulates EMT markers in HNSCC cells

**Fig. 3:**
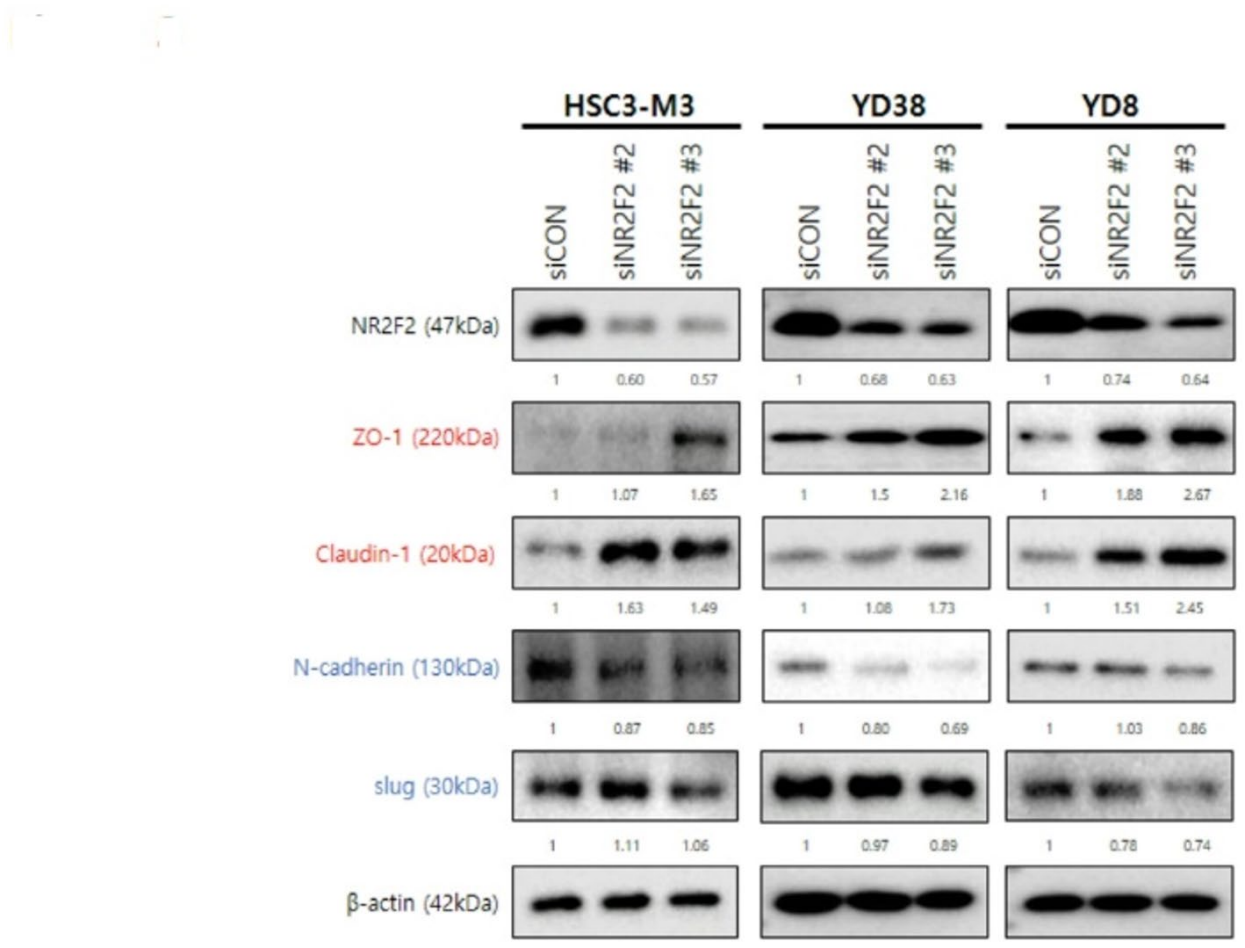
EMT marker regulated by knockdown of NR2F2. Western blotting (WB) analysis of the protein expression of EMT markers in HNSCC cells after NR2F2 siRNA transfection for 24 h. WB analysis showed increased ZO-1 and claudin-1 levels and decreased N-cadherin and slug levels in response to NR2F2 depletion. β-actin was used as an internal loading control

To investigate whether inhibition of NR2F2 regulates EMT markers, we performed a WB analysis. The siRNAs with high efficiency, siNR2F2 #2 and siNR2F2 #3, were transfected into three HNSCC cell lines (HSC3-M3, YD38, and YD8). After 24 h of incubation, protein samples for WB were obtained. As shown in Fig. 3, inhibition of NR2F2 using siNR2F2 resulted in increased expression of epithelial markers (ZO-1 and claudin-1) and decreased expression of mesenchymal markers (N-cadherin and slug) in all three HNSCC cell lines. These findings suggest that knockdown of NR2F2 has the potential to decrease EMT transition and subsequently reduce invasion ability.

### Inhibition of NR2F2 with NR2F2 inhibitor reduces invasion ability and modulates EMT markers in HNSCC cells

To investigate the impact of NR2F2 inhibition on invasion ability and EMT marker modulation, we employed the small molecule CIA as an NR2F2 inhibitor. First, we assessed the cytotoxicity of CIA using an MTT assay (Supplementary Fig. 4). The IC50 of CIA was determined to be 15.61µM in HSC3-M3 cells, 5.57µM in YD38 cells, and 8.49µM in YD8 cells. Subsequently, we used a concentration below 1µM, which showed more than 90% cell viability, for all further experiments.

**Fig. 4:**
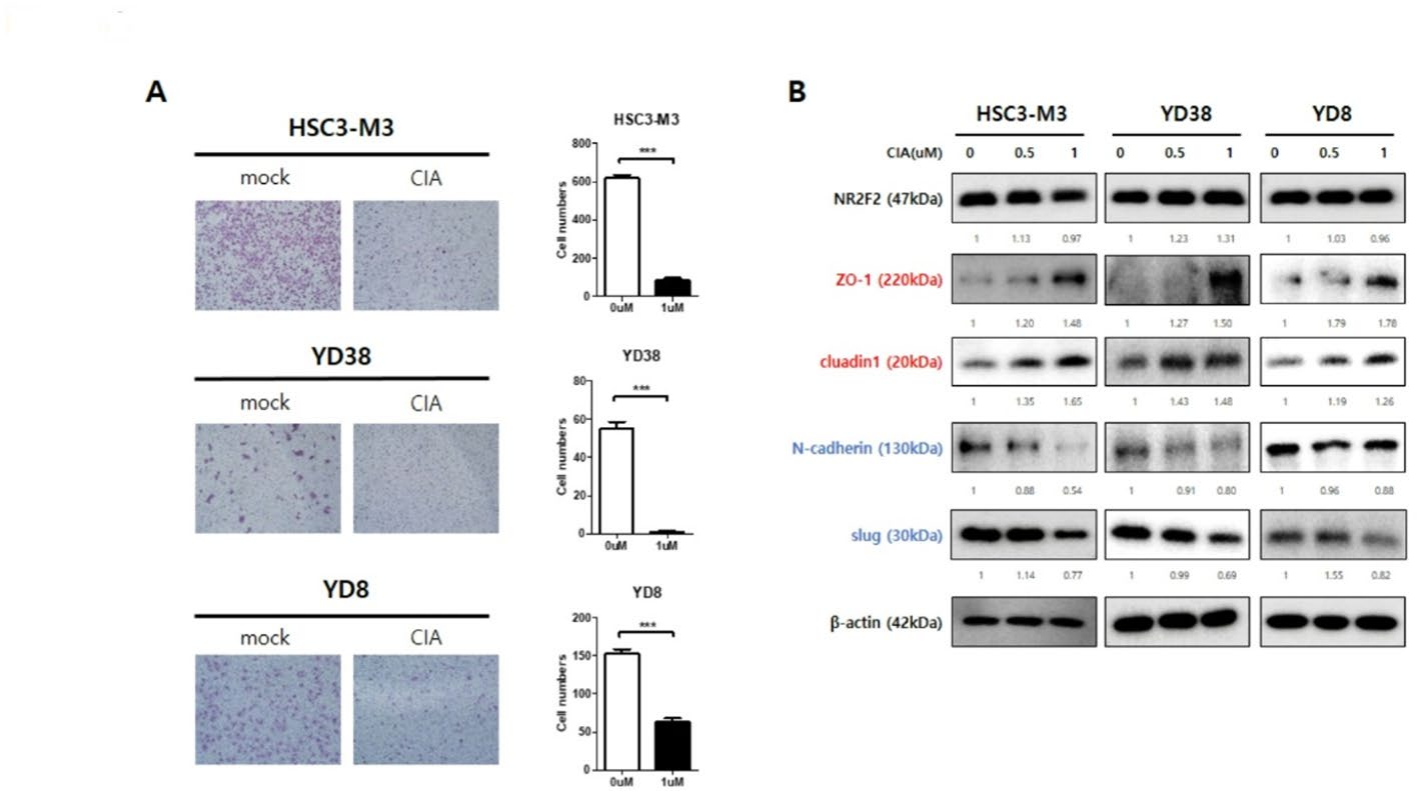
CIA (NR2F2 inhibitor) leads to repression of invasion ability. (A) Representative images show the invasion ability of HNSCC cells with the treatment of 1µM CIA. Statistical significance was determined using a t-test. ***p < 0.005 vs. control. (B) Western blot analysis showed the protein expression of EMT markers in HNSCC cells with the treatment of CIA. β-actin was used as an internal loading control

Treatment with CIA significantly reduced the number of invaded cells in all three HNSCC cell lines (HSC3-M3, YD38, and YD8) (Fig. 4A). This indicates that NR2F2 inhibition through CIA effectively decreases invasion ability in HNSCC cells. Furthermore, we investigated the modulation of EMT markers by NR2F2 inhibition using WB analysis (Fig. 4B). While CIA did not alter the expression of NR2F2, it acted as a molecule that reduced NR2F2 activity. Similar to the results observed with siNR2F2-mediated knockdown, CIA treatment increased the expression of epithelial markers (ZO-1 and claudin-1) and decreased the expression of mesenchymal markers (N-cadherin and slug). Our findings demonstrate that inhibition of NR2F2 using the small molecule CIA significantly reduces invasion ability and modulates EMT markers in HNSCC cells. To explore the effects of NR2F2 inhibition on invasion ability and EMT marker modulation, we utilized the NR2F2 inhibitor 4-MNol. We conducted an invasion assay and WB analysis to assess these effects (Supplementary Fig. 5). Treatment with 4-MNol significantly reduced the number of invaded cells in YD38 and YD8 cells, indicating the impact of NR2F2 inhibition on decreasing invasion ability. Furthermore, the 4-MNol treatment led to an increase in the expression of epithelial markers and a decrease in the expression of mesenchymal markers, demonstrating the modulation of EMT markers. These results suggest the potential of NR2F2 inhibition as a promising therapeutic approach for inhibiting invasion ability and modulating EMT in HNSCC. Further research and evaluation of NR2F2 inhibitors are warranted for their potential application in HNSCC treatment.

## Discussion

NR2F2, a transcription factor belonging to the family of nuclear orphan receptors, is expressed at low levels in the adult heart. However, its expression increases under certain pathological conditions, such as heart failure [21]. Previous studies have shown that elevated NR2F2 expression is associated with mitochondrial dysfunction, increased production of reactive oxygen species (ROS), and changes in energy metabolism [13, 21]. Additionally, NR2F2 has been found to play a role in cell proliferation and immunomodulation in Wharton’s Jelly stem cells (WJ-MSCs), which are multipotent mesenchymal stem cells known for their rapid proliferation and low immunogenicity [22].

In lung cancer-derived brain metastasis, the Wnt/NR2F2/GPX4 pathway has been shown to promote acquired chemoresistance by suppressing ferroptosis through increased consumption of glutathione (GSH) [23]. Previous studies have also implicated NR2F2 in promoting EMT and metastasis in various types of cancer [24, 25]. In gastric cancer (GC), high NR2F2 expression contributed to poor survival [26]. MicroRNA-27b was targeted and down-regulated by NR2F2 in human GC tissues and cells. The ectopic expression of miR-27b inhibited GC cell proliferation and tumor growth in vitro and in vivo by targeting NR2F2. However, the relationship between NR2F2 and HNSCC has not been clearly defined. Our study reveals that NR2F2 plays a role in regulating EMT markers and promoting cell invasion in HNSCC.

The NR2F2 inhibitor, CIA, does not affect the protein levels of NR2F2. Furthermore, it does not interfere with the binding of NR2F2 to its target gene promoters, suggesting that CIA inhibitors do not directly interact with the DNA binding domain of NR2F2 [19]. However, CIA disrupts the interaction between NR2F2 and transcription regulators, including Forkhead box A1 (FOXA1), which leads to the dysregulation of target gene expression and impairs NR2F2 activity. This disruption highlights the significance of the NR2F2 and FOXA1 interaction in gene regulation and suggests that CIA-mediated disruption of this interaction may impact tumor growth. Additionally, FOXA1 has been implicated in regulating EMT independently of androgen receptor (AR) signaling [27]. In our WB results, inhibition of NR2F2 using CIA significantly increased epithelial genes (ZO-1 and claudin1) and decreased mesenchymal genes (N-cadherin and slug). While our results did not directly demonstrate the interaction between NR2F2/FOXA1 and EMT-related genes, our findings suggest a potential role for NR2F2/FOXA1 in regulating EMT-related genes.

4-MNol inhibition of NR2F2 might not rely on a direct blockade of cofactor interaction but could rather stabilize an auto-repressed conformation [20]. 4-MNol, similar to CIA, does not alter the protein levels of NR2F2 but inhibits its activation. Inhibition of NR2F2 using 4-MNol has been shown to decrease the invasion ability of HNSCC cells and regulate the expression of EMT-related genes. Our study also demonstrated the involvement of NR2F2’s auto-repressed conformation in the inhibition of NR2F2, which contributes to the regulation of EMT marker genes and the control of invasion ability.

Previous studies have demonstrated the therapeutic potential of NR2F2 in various cancers. NR2F2 has been found to be overexpressed in pancreatic cancer, where it plays a critical role in angiogenesis and lymphangiogenesis during tumor progression and metastasis [28]. It regulates blood vessel sprouting by influencing endothelial cell proliferation, migration, and VEGF/VEGFR-2 signaling. Also, NR2F2 is found to be expressed in PC and associated with advanced disease stages [29]. Silencing NR2F2 reduces PC cell growth, invasiveness, and angiogenesis. NR2F2 inhibition leads to a significant reduction in tumor growth in vivo. Similarly, NR2F2 has been implicated in promoting tumor invasion and migration in colorectal cancer [30]. It exerts its effects by modulating the expression of EMT-related genes and inhibiting the tumor suppressor miR-34a.

In our study, we focused on the role of NR2F2 in HNSCC. We observed that NR2F2 expression was upregulated in patients with lymph node metastasis, suggesting its involvement in HNSCC progression and metastasis. Further investigation using siRNA and inhibitors of NR2F2 demonstrated that inhibition of NR2F2 significantly decreased the invasion ability of HNSCC cells. Modulation of EMT markers was also observed upon NR2F2 inhibition, supporting the role of NR2F2 in regulating EMT processes in HNSCC. These findings suggest that NR2F2 plays a critical role in promoting invasion and modulating EMT-related processes in HNSCC. Further studies will be needed to explore the underlying mechanisms in greater depth and to validate the therapeutic potential of NR2F2 in appropriate in vivo models.

## Conclusion

Taken together, these findings highlight the therapeutic potential of targeting NR2F2 in cancer therapy. Inhibition of NR2F2 holds promise as a strategy to impair tumor progression, invasion, and metastasis by modulating angiogenesis, lymphangiogenesis, and EMT processes. Further research is needed to elucidate the underlying mechanisms and validate the effectiveness of NR2F2 inhibition as a therapeutic strategy for different cancer types, including HNSCC.

## Supplementary Information

Supplementary Material 1. Fig. S1 (A) GSEA was performed using TCGA HNSCC expression data ranked by correlation with NR2F2 expression. Shown are representative enrichment plots of selected gene sets positively associated with NR2F2-high samples. (B) Scatter plots show Pearson correlation between NR2F2 and canonical epithelial-mesenchymal transition (EMT) markers in TCGA HNSCC cohort (*n* = 520). NR2F2 expression was negatively correlated with CDH1 (epithelial marker) and positively correlated with mesenchymal markers VIM, ZEB1, and TWIST1. Correlation coefficients (r) and p-values are indicated for each comparison.

Supplementary Material 2. Fig. S2. siRNA-mediated knockdown of NR2F2. Western blot analysis demonstrates the depletion of NR2F2 using three siRNA for NR2F2 in YD38 and YD8 cell lines. The remaining siRNAs, excluding #1 siRNA, which showed effective knockdown efficiency, were selected for subsequent experiments.

Supplementary Material 3. Fig. S3. Assessment of NR2F2 knockdown on proliferation. Proliferation assays were conducted in HNSCC cells treated with siCON and siNR2F2.

Supplementary Material 4. Fig. S4. Assessment of cell viability in non-treated and CIA-treated HNSCC cells. MTT assays were performed in HSC3-M3, YD38, and YD8 cell lines to evaluate the effect of CIA on cell viability.

Supplementary Material 5. Fig. S5. Treatment with the NR2F2 inhibitor, 4-MNol, resulted in significant repression of invasion ability in both YD38 and YD8 cell lines. (A) Representative images demonstrate the invasion ability of the cell lines after treatment with 0.1µM 4-MNol. Statistical significance was determined using a t-test. ***p < 0.05 vs. control. (B) Western blot analysis was conducted to assess the protein expression of EMT markers in the YD38 and YD8 cell lines following 4-MNol treatment. β-actin was used as an internal loading control.

## Data Availability

The RNA-seq datasets analysed during the current study are publicly available in The Cancer Genome Atlas (TCGA) repository, https://portal.gdc.cancer.gov/.

## Abbreviations

HNSCC: Head and neck squamous cell carcinoma
NR2F2: Nuclear receptor subfamily 2 group F member 2
COUP-TF: Chicken ovalbumin upstream promoter transcription factor
SCC: Skin squamous cell carcinoma
EMT: Epithelial-mesenchymal transition
TCGA: The Cancer Genome Atlas
CIA: COUP-TFII inhibitor A
4-MNol: 4-methoxynaphthalene-1-ol
LNM: Lymph node metastasis
ROS: Reactive oxygen species
WJ-MSCs: Wharton’s jelly stem cells
GSH: Glutathione
GC: Gastric cancer
FOXA1: Forkhead box A1
