# Supplementary figures and images for: Inhibition of NR2F2 suppresses invasion ability and modulates EMT marker in head and neck squamous cell carcinoma
Source: Discov Oncol. 2025 Oct 15;16:1887. doi: 10.1007/s12672-025-03539-3 (PMC12528631; doi:10.1007/s12672-025-03539-3)

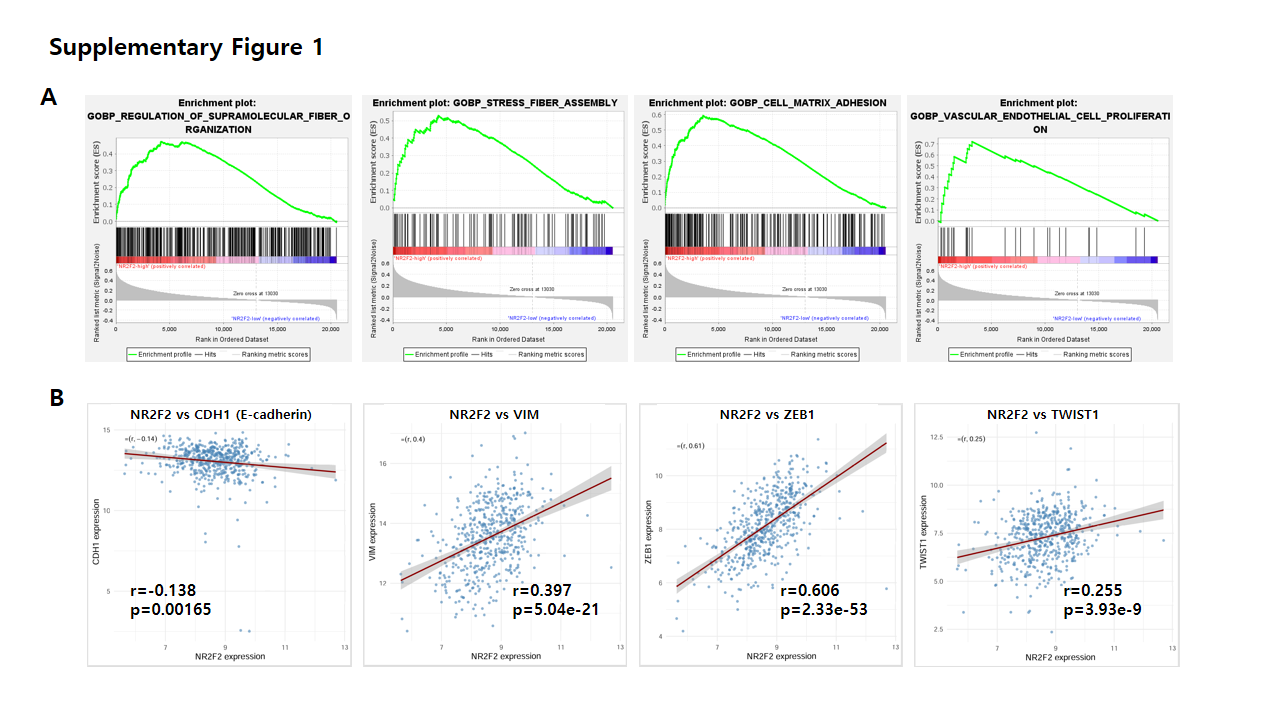

Supplement: Supplementary file 1 — Supplementary Material 1. Fig. S1 (A) GSEA was performed using TCGA HNSCC expression data ranked by correlation with NR2F2 expression. Shown are representative enrichment plots of selected gene sets positively associated with NR2F2-high samples. (B) Scatter plots show Pearson correlation between NR2F2 and canonical epithelial-mesenchymal transition (EMT) markers in TCGA HNSCC cohort (n = 520). NR2F2 expression was negatively correlated with CDH1 (epithelial marker) and positively correlated with mesenchymal markers VIM, ZEB1, and TWIST1. Correlation coefficients (r) and p-values are indicated for each comparison. [file 12672_2025_3539_MOESM1_ESM.tif]

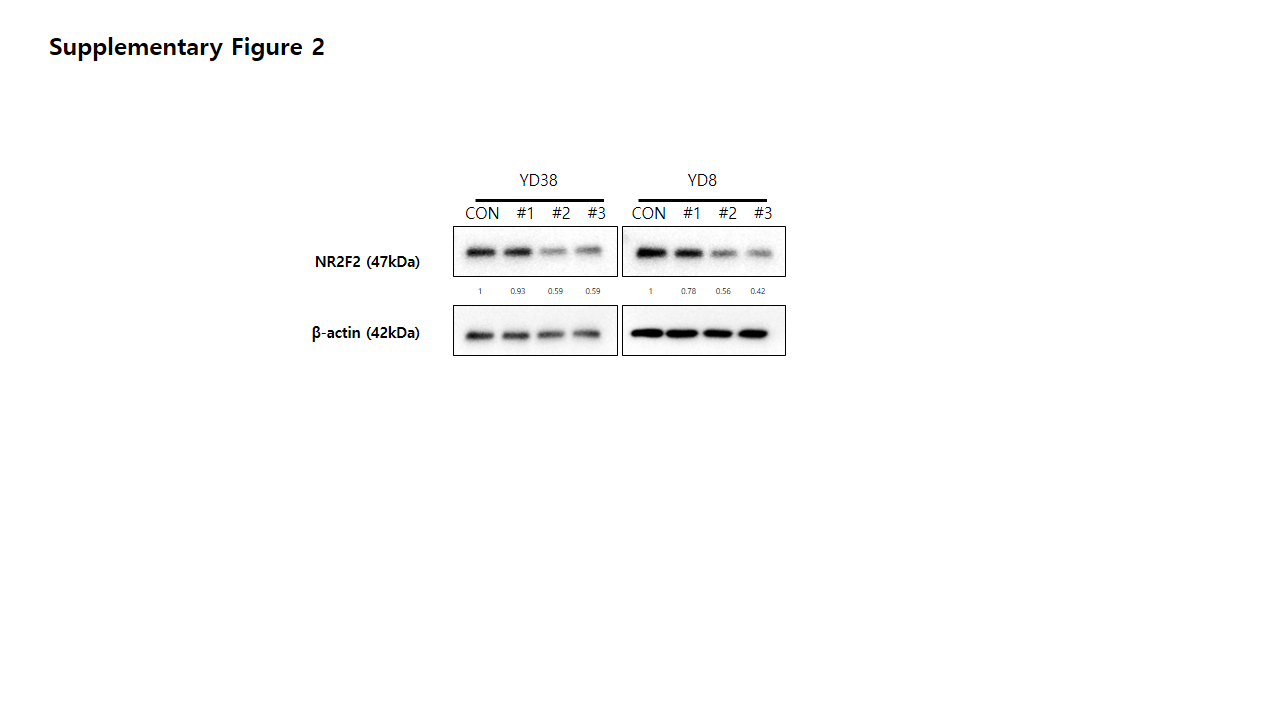

Supplement: Supplementary file 2 — Supplementary Material 2. Fig. S2. siRNA-mediated knockdown of NR2F2. Western blot analysis demonstrates the depletion of NR2F2 using three siRNA for NR2F2 in YD38 and YD8 cell lines. The remaining siRNAs, excluding #1 siRNA, which showed effective knockdown efficiency, were selected for subsequent experiments. [file 12672_2025_3539_MOESM2_ESM.tif]

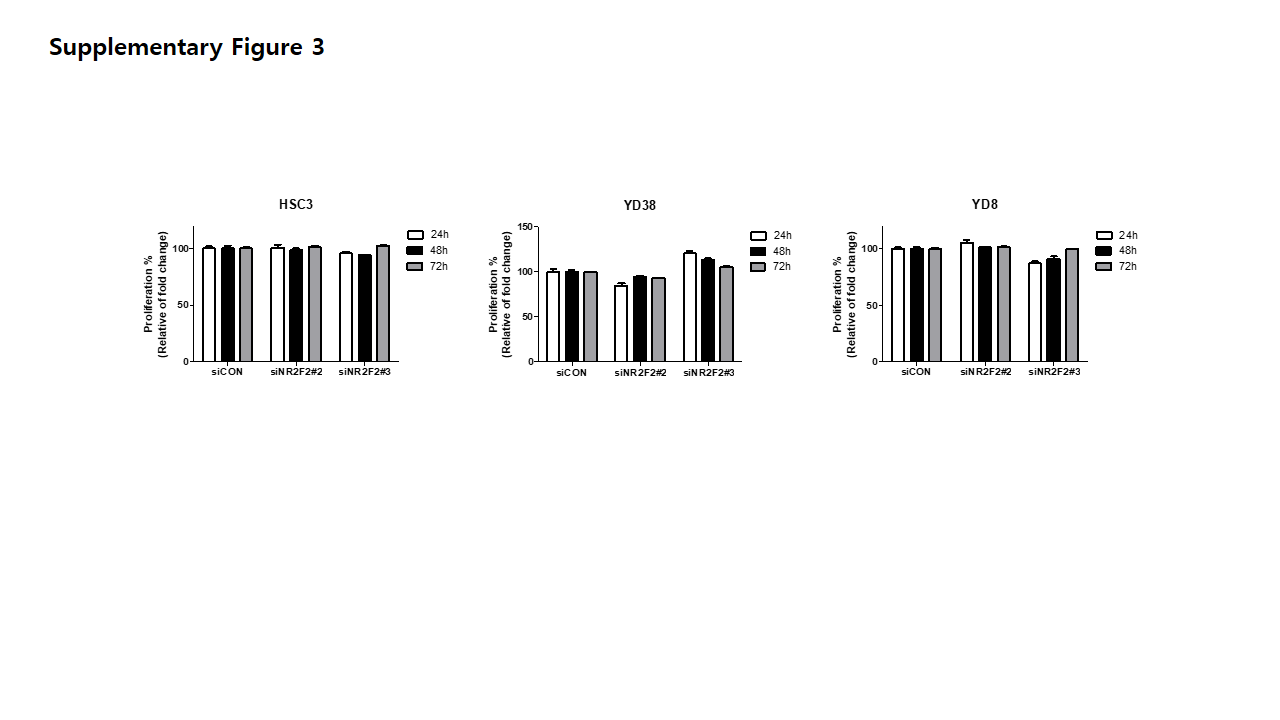

Supplement: Supplementary file 3 — Supplementary Material 3. Fig. S3. Assessment of NR2F2 knockdown on proliferation. Proliferation assays were conducted in HNSCC cells treated with siCON and siNR2F2. [file 12672_2025_3539_MOESM3_ESM.tif]

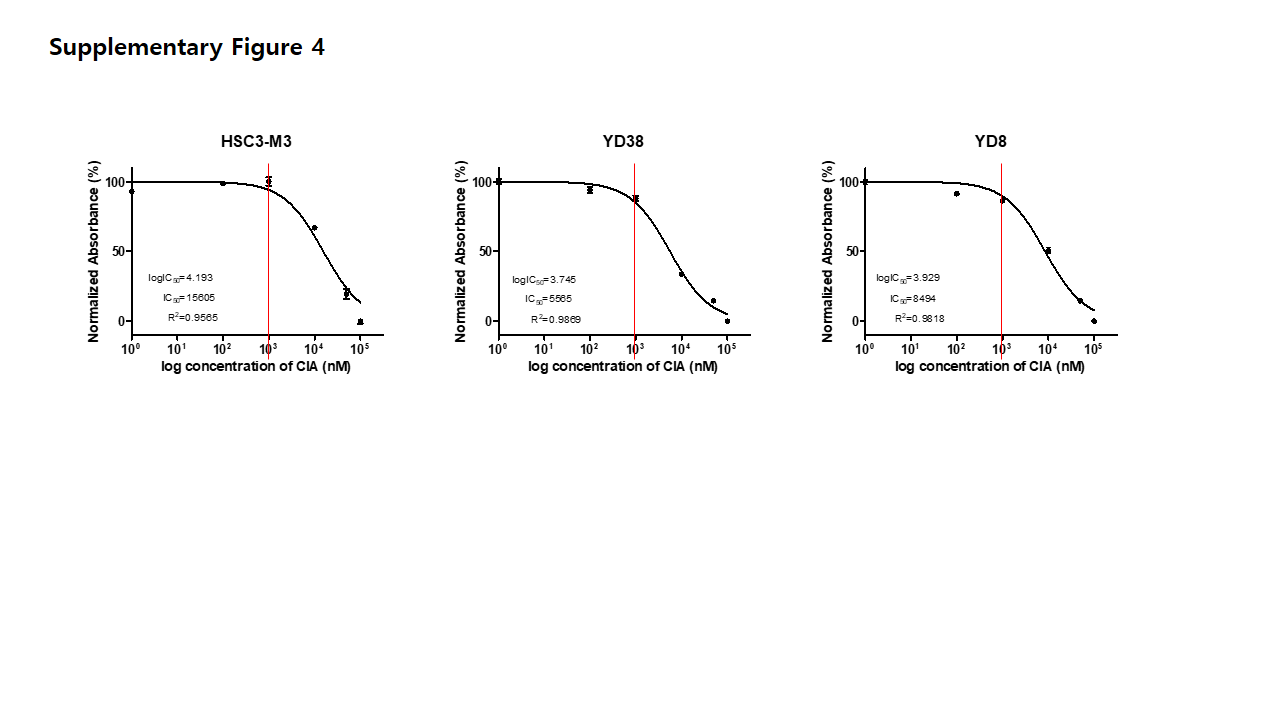

Supplement: Supplementary file 4 — Supplementary Material 4. Fig. S4. Assessment of cell viability in non-treated and CIA-treated HNSCC cells. MTT assays were performed in HSC3-M3, YD38, and YD8 cell lines to evaluate the effect of CIA on cell viability. [file 12672_2025_3539_MOESM4_ESM.tif]

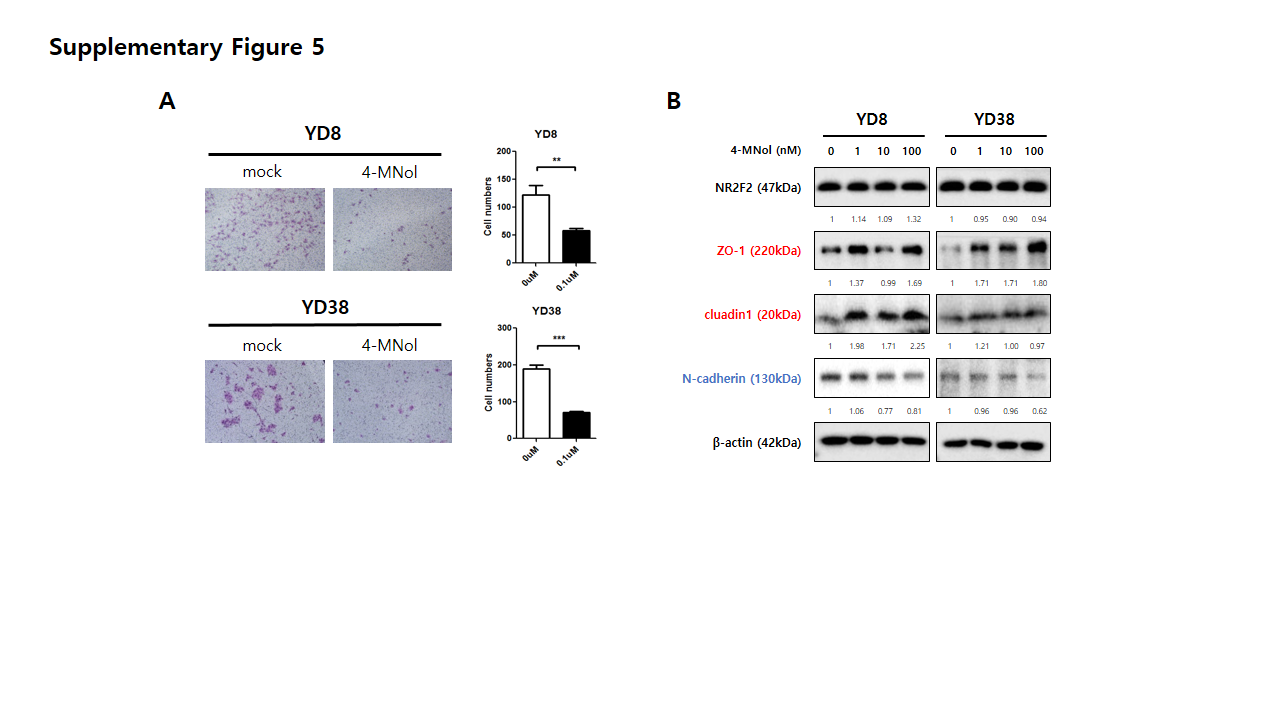

Supplement: Supplementary file 5 — Supplementary Material 5. Fig. S5. Treatment with the NR2F2 inhibitor, 4-MNol, resulted in significant repression of invasion ability in both YD38 and YD8 cell lines. (A) Representative images demonstrate the invasion ability of the cell lines after treatment with 0.1µM 4-MNol. Statistical significance was determined using a t-test. ***p < 0.05 vs. control. (B) Western blot analysis was conducted to assess the protein expression of EMT markers in the YD38 and YD8 cell lines following 4-MNol treatment. β-actin was used as an internal loading control. [file 12672_2025_3539_MOESM5_ESM.tif]
